# Detection and validation of single feature polymorphisms in cowpea (*Vigna unguiculata *L. Walp) using a soybean genome array

**DOI:** 10.1186/1471-2164-9-107

**Published:** 2008-02-28

**Authors:** Sayan Das, Prasanna R Bhat, Chinta Sudhakar, Jeffrey D Ehlers, Steve Wanamaker, Philip A Roberts, Xinping Cui, Timothy J Close

**Affiliations:** 1 Department of Botany and Plant Sciences, University of California, Riverside, CA 92521 USA; 2 Department of Nematology, University of California, Riverside, CA 92521 USA; 3 Department of Botany and Biotechnology, Sri Krishnadevaraya University, Anantapur, 515 003 India; 4 Department of Statistics, University of California, Riverside, CA 92521 USA

## Abstract

**Background:**

Cowpea (*Vigna unguiculata *L. Walp) is an important food and fodder legume of the semiarid tropics and subtropics worldwide, especially in sub-Saharan Africa. High density genetic linkage maps are needed for marker assisted breeding but are not available for cowpea. A single feature polymorphism (SFP) is a microarray-based marker which can be used for high throughput genotyping and high density mapping.

**Results:**

Here we report detection and validation of SFPs in cowpea using a readily available soybean (*Glycine max) *genome array. Robustified projection pursuit (RPP) was used for statistical analysis using RNA as a surrogate for DNA. Using a 15% outlying score cut-off, 1058 potential SFPs were enumerated between two parents of a recombinant inbred line (RIL) population segregating for several important traits including drought tolerance, *Fusarium *and brown blotch resistance, grain size and photoperiod sensitivity. Sequencing of 25 putative polymorphism-containing amplicons yielded a SFP probe set validation rate of 68%.

**Conclusion:**

We conclude that the Affymetrix soybean genome array is a satisfactory platform for identification of some 1000's of SFPs for cowpea. This study provides an example of extension of genomic resources from a well supported species to an orphan crop. Presumably, other legume systems are similarly tractable to SFP marker development using existing legume array resources.

## Background

Cowpea (*Vigna unguiculata *L. Walp) is grown extensively as a food and fodder crop in West Africa, lower elevation areas of eastern and southern Africa, north-eastern Brazil, parts of the Middle East, India, and the south-eastern and south-western regions of North America [1]. Like common beans (*Phaseolus vulgaris *L.) which are combined with maize or other starchy staple crops in other parts of the world, dry grain cowpea is consumed with lower protein cereal and root/tuber staples to provide an adequate protein quantity and quality to hundreds of millions of rural and urban consumers in West Africa [2,3]. Cowpea forage is used for livestock and cowpea hay plays a critical role as fodder during the dry season in West Africa [4]. 'Longbean' or 'Asparagus bean' of cowpea cultivar group Sesquipedialis is considered one of the top-ten Asian vegetable crops and is grown on at least 400,000 hectares worldwide for production of fresh 'green' or 'snap' beans.

Cowpea (2n = 2x = 22) with genome size ~600 Mb belongs to the genus *Vigna *Savi. (subgenus *Vigna *sect. Catiang) in the Phaseoleae [5]. Genomic resources such as cDNA libraries, ESTs and BAC libraries have been meagre in cowpea [6] until very recently. High-resolution genetic maps provide breeders the ability to analyze the inheritance of genes of interest, monitor the transmission of specific genes or genomic regions from parents to progeny, and accelerate map-based cloning [7,8]. However, relatively few genetic resources are available to cowpea breeders, and molecular marker-based selection is only possible for a few traits [6,9-12]. Efforts made previously for linkage mapping in cowpea include 92 RFLP markers [13], 181 markers that are mostly RAPDs [14], and 242 markers that are mostly amplified fragment length polymorphisms (AFLP) together with 17 biological resistance traits and resistance gene analogs [9]. Currently available genetic maps for cowpea are of limited utility for breeders due to the lack of markers tightly linked to important traits such as root-knot nematode resistance [10].

Any marker system with increased throughput, decreased cost per data-point, and greater map resolution is highly desirable [7,15] for genetic mapping and marker assisted breeding. Oligonucleotide-based microarrays have been used in recent years to identify genetic polymorphisms [16]. Winzeler *et al*. [17] first reported the hybridization of labelled genomic DNA to oligonucleotide microarrays to identify sequence polymorphisms in haploid yeast. Borevitz *et al*. [18] coined the term "single feature polymorphism" and demonstrated that this approach can be applied to organisms with somewhat larger genomes, specifically *Arabidopsis thaliana *with a genome size of 140 Mb. Similarly, whole-genome DNA-based SFP detection has been accomplished in rice [19], which has a genome size of 440 Mb, though with a higher false discovery rate, as will be discussed later. For barley, which has a 5300 Mb genome composed of more than 90% repetitive DNA, Cui *et al*. [20] and Rostoks *et al*. [21] hybridized the Affymetrix Barley1 expression microarray with RNA-derived cRNA to reduce the target complexity, enabling detection of some thousands of SFPs. Array-based genotyping by hybridizing with cRNA instead of DNA was initially accomplished in yeast [22], and subsequently in *Arabidopsis *[23] following the work cited above in barley. SFPs have been used for genome-wide association mapping and linkage disequilibrium studies [24], and to estimate mutation and recombination parameters in populations [25]. Thus SFPs have become an attractive marker system for various applications including parental polymorphism discovery, which is the present subject of our work on cowpea.

Cowpea has been identified as an "orphan crop" recommended for increased support for biotechnology research [26]. There are many opportunities to apply knowledge from "model species" such as *Arabidopsis*, rice (*Oryza sativa*), and *Medicago truncatula *to crops like cowpea. Relatively large genetic gains can be expected from investments in applied plant breeding in cowpea [6].

SFP's based on expressed sequences are an efficient source of large number of genic markers in cowpea relative to moderate-throughput and usually non-genic marker systems such as RAPDs, AFLPs and SSRs. Here we show that the Affymetrix soybean genome array is a satisfactory system for SFP discovery in cowpea, which belongs to the same family as soybean. From this observation, we claim that SFPs can also be identified efficiently for other "orphan" legumes using existing genome arrays for soybean or *Medicago truncatula*.

## Results

### Cross species platform for array hybridization

When cowpea cRNAs were hybridized to the soybean genome array, the frequency of "present" calls ranged from 11 to 14.7% of all probe sets on the array (see Methods for the definition of "present" call). This is a frequency of 18 to 24.7% of probe sets specific to soybean (Table 1) since only 61% of the probe sets target soybean genes (see Methods).

**Table 1:** Percent present calls from cowpea transcript hybridization to soybean genome array.

Genotype	Treatment	Replicate	% Present call*	% Present call* *Glycine max*
CB46	unstressed	1	14.7	24
CB46	unstressed	2	13.9	22.8
CB46	stressed	1	11.5	18.7
CB46	stressed	2	12.5	20.4
IT93K-503-1	unstressed	1	14.3	23.4
IT93K-503-1	unstressed	2	15.2	24.7
IT93K-503-1	stressed	1	13.9	22.8
IT93K-503-1	stressed	2	11	18

		Range	11 – 14.7	18 – 24
		Mean	13.4	21.9

*See Methods for definition of "present call"

### SFP detection and validation

Robustified projection pursuit (RPP) [20] was used for SFP detection between two parental genotypes (CB46 and IT93K-503-1) of a RIL population segregating for several important agronomic traits. As stated in Methods, RPP provides a list of SFP probe sets and single probes positioned directly over genetic polymorphisms. Because the RPP method depends on the availability of "present" calls in both of the comparison genotypes, and because only about 20% of the probe sets are called "present" using cowpea RNA, only about 7,000 of the 37,500 soybean probe sets have the potential to detect SFPs in cowpea from typical cowpea RNA samples. Using a top 15% outlying score cut-off, we generated a list of 1058 putative SFP probes between genotypes CB46 and IT93K-503-1. A full list of these SFPs and their outlying scores is provided in Additional file 1. The question then was, "Do these statistically detected SFPs represent real genetic polymorphisms in cowpea?"

**Figure 1:**
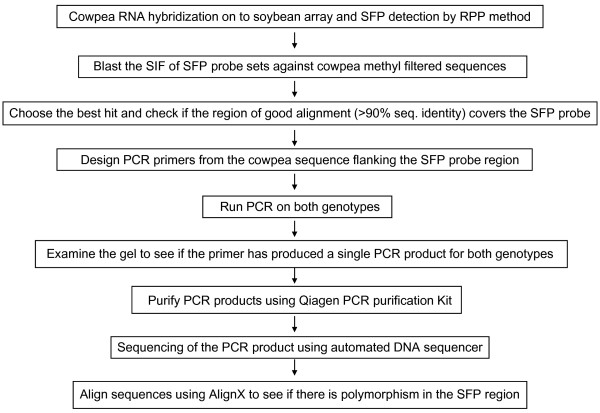
Schematic diagram of SFP validation protocol.

**Figure 2:**
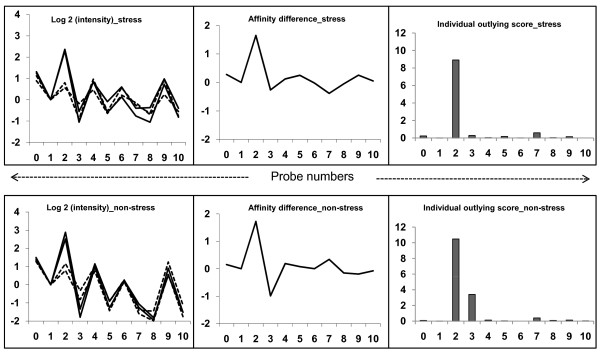
**Plots of signal intensities, affinity differences and individual outlying scores**. Left panels: log intensities (PM, perfect match) for a representative probe set (Gma.1863.1.S1_at) from two genotypes. Middle panels: the differences of average log intensities between two genotypes. Right panel: individual outlying scores for each probe. Dotted lines indicate IT93K-503-1 and solid lines indicate CB46. This SFP was identified both in stress (upper layer) and non-stress (lower layer) datasets.

A schematic diagram of SFP detection and validation is given in Figure 1. Plots of the log intensities, affinity differences and individual outlying scores for a representative probe set (Gma.1863.1.S1_at) under both drought-stress and non-stress conditions are shown in Figure 2. The intensity differentiation is highest at probe 2 between CB46 and IT93K-503-1, indicating polymorphism at this probe position [20]. Probe 2 was selected as the best SFP probe by the RPP method based on having the highest outlying score.

### PCR and gel electrophoresis

**Figure 3:**
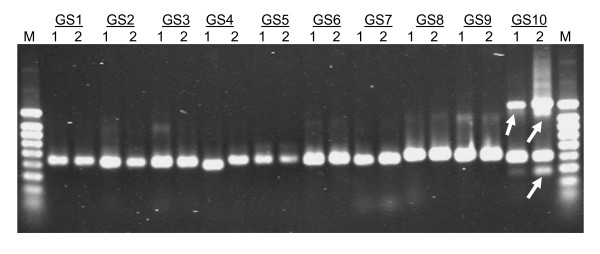
**A representative gel image of PCR amplification**. Aliquots of the PCR products of ten (GS1 to GS10) representative primer pairs were loaded and separated on a 1.2% agarose gel. 1, CB46; 2, IT93K-503-1; M, size marker. The arrows indicate non-specific amplification.

A list of primers used and the expected and observed PCR amplicon sizes are provided in Table 2. PCR amplicon sizes ranged from 400 – 450 bp. A representative gel image of PCR amplification is shown in Figure 3. For the nine primer pairs (GS1 to GS9), the amplicon sizes were as predicted by Primer3 (see Methods), the primer designing software. For primer pair GS 10, non specific bands were detected in both genotypes. For this primer pair the 'T_m_' was increased during PCR but still no specific band of the predicted size was amplified. Primer pairs yielding such anomalies, or which yielded no amplicon from one or both cowpea genotypes (not shown), were not pursued with amplicon sequencing. For calculation of the validation rate, the numerator is the number of amplicons containing a polymorphism within the region spanned by a SFP probe, and the denominator is the number of amplicons from which sequences were generated.

**Table 2:** Primer list and amplicon lengths for 17 validated SFP-containing amplicons.

**Affymetix probe set**	**Primer Name**	**Forward primer (5' – 3')**	**Reverse Primer (5 – 3')**	Predicted* amplicon size (bp)	**Observed^# ^amplicon size (bp)**
Gma.7528.1.S1_s_at	GS01	GTACCTTCCGGTGGATTCAA	TGGAATGATCACTTGGCAGA	435	439
GmaAffx.93250.1.S1_s_at	GS02	AATCCGTGTGCCAGGAATAG	ACCCAATCAGACCAATGGAG	416	420
Gma.1164.2.S1_at	GS03	GTCTTTTTGGCCTTGCATGT	TCTCAGCTTCTCCGTCCATT	422	424
Gma.1555.1.S1_a_at	GS04	AACAACAGAGTGAGCCAGCA	ACCAGGGTACCTCCCTTGAC	400	402
Gma.1863.1.S1_at	GS07	GCAGGAGTGCGTTTACCCTA	TGCTTTCACACCGCAAATTA	417	424
GmaAffx.86591.1.S1_at	GS08	TGGGAGCTGTGTCAACAGAA	GGCCTCTCAAACTGAACGTC	431	433
Gma.4431.1.S1_at	GS12	TAGCAGCCAGCCTGTGTATG	CGAAGGGTCCTAAACAACCA	447	449
Gma.8053.1.S1_at	GS16	ATCTGAGGCAGCAGCAAAAG	TGCCATGGCCACTTTAGATT	402	407
GmaAffx.62051.1.S1_at	GS21	GAAGCGTTGCATGCTTATCC	CATTCCAGTCACACCACCAG	429	432
GmaAffx.58155.1.S1_at	GS22	GTTAAACGCACCGATGGACT	ACACACTCGCCAAACAATGA	404	406
Gma.5674.1.S1_a_at	GS23	GCGGTGTTTCTTTCATGGTT	TCCCTCGTATATTCGGCATT	429	433
Gma.1449.1.S1_s_at	GS24	GCCTTTCTTCAGTGGATTGG	TGATTCACAACCCCATTTGA	442	444
Gma.13293.1.S1_at	GS27	TCTGCATTAAGCCACTGCAC	AATAGCAGCACCACGATTCC	442	441
GmaAffx.14067.1.S1_at	GS29	GGCATCCCTCTCAAGAATGT	GCAACAAAAATGGGGTGAGA	424	427
GmaAffx.69322.1.S1_at	GS31	AGTCTTATGTTGGCACAAAAACA	GCCAACTCTACCCACCAAGA	433	433
GmaAffx.28120.1.s1_at	GS36	CATCAGACACAGACGGCACT	TCACACCAATCTCCCAAACA	414	413
GmaAffx.70836.1.S1_at	GS37	CGTTCCAGTGGACATTATGC	AGATTCTTTTTGCCCAAGCA	441	431

*Predicted size indicates the PCR product size anticipated by the primer3 program.

^#^Observed size indicates size of amplicon sequences after assembling the bidirectional sequence reads.

### Alignment of amplicon sequences for SFP validation

**Figure 4:**
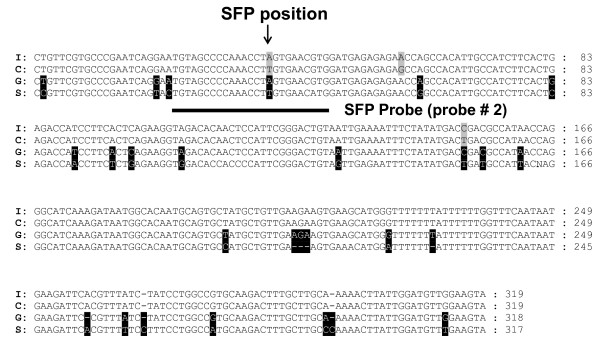
**Alignment of cowpea amplicon sequences related to a probe set (Gma.1863.1.S1_at) and its target sequence from the soybean SIF**. Polymorphic residues between CB46 and IT93K-503-1 are highlighted in grey, polymorphic residues between cowpea methyl filtered sequence and soybean SIF are in black. The position of SFP probe number 2 detected by the RPP method is underlined. Arrows indicate SNPs. I, IT93K-503-1; C, CB46; G, cowpea methyl filtered sequence; S, target sequence from soybean SIF.

**Figure 5:**
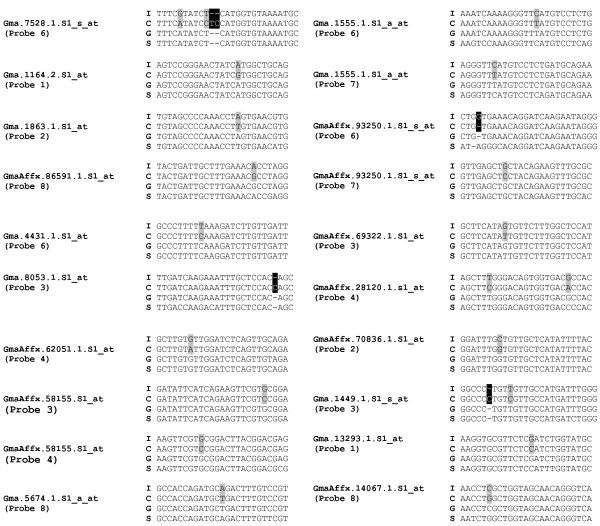
**Alignments of validated SFPs**. Grey background indicates SNP and black background indicates INDEL. I, IT-93K-503-1; C, CB46; G, methyl filtered cowpea sequence; S, target sequence from soybean SIF. The target SFP probe number is given in parenthesis.

To estimate the SFP validation rate we selected 25 putative SFP probe sets. A total of 17 (68%) of these 25 SFP probe sets were validated by amplicon sequences. A representative alignment of genomic amplicon sequences with the target sequence of probe set Gma.1863.1.S1_at is shown in Figure 4. Probe position 2 spans a polymorphism (a SNP in this case), as predicted by the RPP method. Alignments of the 20 validated SFP probes from the 17 validated probe sets (three probe sets each contained two SFP probes) are shown in Figure 5. Among these 20 validated SFP probes, 15 SFP (75%) were positioned over a single SNP, 2 (10%) were positioned over only a single nucleotide insertion or deletion (INDEL), 1 (5%) spanned one SNP and one single nucleotide INDEL, 1 (5%) spanned two SNPs, and 1 (5%) spanned two SNPs and a dinucleotide INDEL.

## Discussion

### Array-based genotyping

The availability of arrays designed to measure gene expression for a wide range of species has spawned considerable interest in identifying single feature polymorphisms (SFPs) from DNA hybridization and transcriptome data [27]. SFPs derived from transcript sequences provide a link to structural genes and are particularly attractive for species which lack sequence resources for marker development. The RPP method [20] is distinct from several commonly used statistical approaches to SFP identification [27] in that the RPP method does not accept expression level polymorphisms, which may be attributed to linked cis-acting regulatory regions or to unlinked trans-acting factors. Instead, RPP limits its SFP calls to those that are most likely to be positioned over a genetic polymorphism. This maximizes the relationship between the probe set functional annotation and the gene containing the detected polymorphism, which consequently maximizes the potential for cross-species synteny mapping using SFPs. A simplification of the RPP method has been used for 16,000 Mb hexaploid wheat to map markers termed "high variance probe sets" to delimit the positions of translocation breakpoints using wheat-rice synteny [28]. Here we report cross species application of the Affymetrix soybean genome array by RPP for polymorphism identification in parents of a cowpea recombinant inbred line mapping population.

The false positive rate in SFP detection is an important consideration for application of SFPs to genetic mapping. The false positive rate is essentially the percentage of statistically defined SFPs that are not valid at the DNA sequence level. Ideally, the false positive rate would be 100% minus the validation rate from amplicon sequencing. However, for cowpea and other species which lack complete genome sequence information, incomplete knowledge of genes, and gene families in particular, limits the validation rate to a theoretical maximum less than 100%. This is because PCR primers for amplicon production must be designed only from known sequences and therefore cannot always target the same gene detected by a SFP probe. Amplification of the wrong member of a gene family bearing no polymorphism can result in a false non-validation of a SFP (false negative). We had access to sequences of less than 70% of all cowpea expressed genes, and therefore also to less than 70% of the members of any given multigene family. This absence of sequence information would tend to cause an overestimation of the false positive rate. However, the selection of SFP probe sets for validation was somewhat skewed toward SFPs with higher outlying scores, which may cause an overestimation of the validation rate. Therefore, the estimated 68% validation rate and inferred 32% false positive are only rough estimates. We have observed that at least 50% of the non-validated cowpea SFPs are in genes that are multi-gene family members. An extreme case of multigene families comes from a study of *Medicago truncatula *nodules, which revealed the *NCR *(nodule-specific cysteine rich) gene family with more than 300 members [29].

A false positive rate of about 5% in SFP detection was reported in yeast [17] and 3% in *Arabidopsis *[18] using genomic DNA. In contrast, approximately a 25% false positive rate was reported in 440 Mb rice using genomic DNA [19], and 10–20% in 5300 Mb barley using RNA-based datasets and RPP [20].

### Cross-species platform for orphan crops/organisms

Recently a number of interspecies comparisons of gene expression have been carried out including human versus monkeys [30,31], between rodents [32], human versus mouse [33], within *Xenopus *[34] and within *Drosophila *[35]. Cross-species analysis of gene expression in non-model mammals was reported by Nieto-Díaz *et al*. [36]. The reproducibility of probe data obtained from hybridizing deer, Old-World primates, and human RNA samples to the Affymetrix human GeneChip^® ^U133 Plus 2.0 was compared. Cross-species hybridization affected neither the distribution of the hybridization reproducibility among different categories nor the reproducibility values of the individual probes. Studies such as these encouraged us to extend the cross-species concept to SFP detection for cowpea using the readily available Affymetrix soybean genome array.

**Figure 6:**
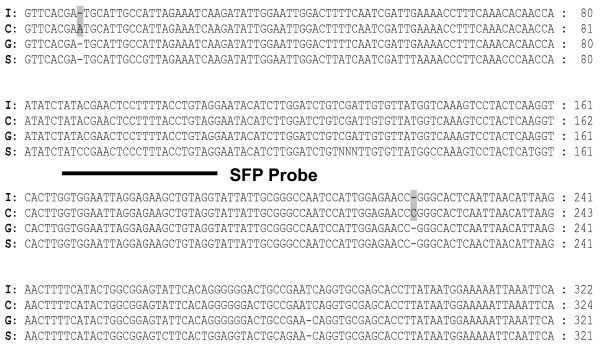
**Useful byproducts of genomic amplicon sequencing**. Residues highlighted in grey indicate polymorphism between two cowpea genotypes though no polymorphism was detected in the SFP probe region detected by RPP (underlined). I, IT93K-503-1; C, CB46; G, cowpea methyl filtered sequence; S, target sequence from soybean SIF.

In the SFP validation process we identified polymorphic sites including SNPs and INDELs in sequences neighbouring the SFP probe positions. Such polymorphisms, which generally represent haplotypes, also can be used for mapping and therefore are useful by-products of genomic amplicon sequencing for SFP validation. As shown in Figure 6, there are even instances of amplicon sequence polymorphisms when no polymorphism is detected in an SFP position. In general cowpea seems to have abundant genetic polymorphism.

### Cost of array-based genotyping

The costs of marker development and application are important to consider. At our cost of $600 per soybean genome array for purchase, labelling, hybridization and data collection, the array-related costs of genotyping 100 RILs from two cowpea genotypes using one array per RIL would be $60,000 USD. If this would yield 1000 mapped SFP markers, then this would amount to $60 USD per mapped marker. Depending on the circumstances, this may or may not be attractive relative to other marker options, and array costs may vary at different locations. Nevertheless, $60 USD per mapped SFP in a 100-RIL population may be useful as an approximate point of reference. Alternatively, using RPP one could readily generate a short list of SFP-bearing probe sets using datasets derived just from parental genotypes and then develop smaller, less expensive arrays containing only the probe sets that contain SFPs, reducing the cost per mapped SFP accordingly.

Another possible application of cross-species SFP analysis would be simply to compare any two genotypes within a species in order to focus amplicon sequence-based marker development on a set of target sequences that have a high chance of revealing a polymorphism. A modest expenditure to produce two datasets from each genotype of interest could provide a sizeable cost savings in materials and labour by increasing the success rate of amplicon sequencing in search of polymorphisms.

## Conclusion

We conclude that the Affymetrix soybean genome array is a satisfactory platform for identification of SFPs in cowpea. This study demonstrated an efficient way to generate genetic SFP markers for orphan crops by using the two parents of a RIL population segregating for several important agronomic traits. SFPs between these two genotypes can be used for high density mapping and those which are tightly linked to phenotypes such as drought or insect tolerance could be used for marker-assisted breeding.

## Methods

### Plant materials

The genotypes used in this study were California Blackeye No. 46 (CB46) and IT93K-503-1. CB46 was developed at the University of California, Davis, released in 1989 [37] and is currently the most widely grown blackeye-type cowpea cultivar in the United States. IT93K-503-1 is a breeding line developed by the International Institute of Tropical Agriculture (IITA) in Ibadan, Nigeria to produce high grain yield in the savanna agro-ecological zone of West Africa. The parental genotypes are inbred pure lines and differ in a number of important agronomic traits including grain size, photoperiod sensitivity, seedling drought tolerance, and resistance to *Fusarium *wilt race 4 and brown blotch fungal diseases. In order to determine the inheritance of these traits and to develop nearby markers, a RIL population with 135 lines was created.

### Soybean genome array

Phylogenetic relationships based on the conserved sequences within Papilionoideae legumes imply that *Vigna *(cowpea) is closely related to soybean [38]. Since a cowpea genome array was not available, a soybean genome array was used to identify SFPs in cowpea. The soybean genome array contains 37,500 probe sets derived from soybean (*Glycine max *L.) unigenes. This represents 61% of the total probe sets on the chip, with the remainder targeting two pathogens important for soybean research, of which 15,800 (26%) probe sets target *Phytophthora sojae *(a water mold) and 7,500 (12%) probe sets target *Heterodera glycines *(soybean cyst nematode). This array uses probe sets composed of 11 probe pairs to measure the expression of each gene. Each probe pair consists of a perfect match (PM) probe and a mismatch probe.

### RNA purification

Seeds were germinated in two sets of pots and grown in controlled glasshouse conditions during spring 2005 under natural photoperiod. The temperature was maintained at day/night cycle of 35/18°C. The growing axillary buds were harvested 14 days after planting (DAP) from one set of pots to provide tissues for control samples (flash frozen and stored at -80°C). Plants in the second set of pots were exposed to drought stress induced by withholding water after germination. The growing axillary buds were harvested from drought stressed seedlings at 14 DAP (flash frozen and stored at -80°C). With two genotypes, two treatments and two replicates, there were a total of 8 samples. Total RNA was isolated using TRIzol (Gibco BRL Life Technologies, Rockville, MD) reagent. RNA was further purified using an RNeasy spin column (Qiagen, Chatsworth, CA) and an on-column DNase treatment. RNA integrity was assessed prior to target preparation using RNA Lab-On-A-Chip (Caliper Technologies Corp., Mountain View, CA) evaluated on an Agilent Bioanalyzer 2100 (Agilent Technologies, Palo Alto, CA).

### Labelling and hybridization of cowpea RNA on soybean genome array

Cowpea RNA samples were used to make biotin tagged cRNAs. These were hybridized to an Affymetrix soybean genome array as recommended by Affymetrix (Affymetrix GeneChip Expression Analysis Technical Manual; Affymetrix Inc., Santa Clara, CA) at the Institute for Integrative Genome Biology Microarray Facility at the University of California, Riverside. The hybridization data were scanned for visible defects and then extracted using default settings and tabulated as CEL files using Affymetrix GeneChip Operating Software (GCOS 1.2). A global scaling factor of 500, a normalization value of 1, and default parameter settings were used for the soybean genome array. The detection calls (present, absent, or marginal) for the probe sets were made by GCOS as follows (abridged from the Affymetrix GeneChip Expression Analysis Technical Manual; Affymetrix Inc., Santa Clara, CA). The detection algorithm uses probe pair intensities to generate a detection *p*-value and assign a "present", "marginal", or "absent" call. Each probe pair in a probe set has a potential vote in determining whether the measured transcript is or is not "present". The vote is described by the discrimination score (R). R is calculated for each probe pair and compared to a predefined threshold, Tau. Probe pairs with R higher than Tau vote "present" and the voting result is summarized as a *p*-value. The greater the number of discrimination scores (R) that are above Tau, the smaller the p-value and the more likely the given transcript is truly present in the sample.

### Method for identifying SFPs

Expression data were generated by hybridizing cowpea cRNA to the soybean genome array. A statistical method called robustified projection pursuit (RPP) was used for SFP analysis [20]. Only the values from the PM probes were utilized. The use of RNA as a surrogate for genomic DNA eliminated interference from highly repetitive DNA as a technical impediment to SFP detection. An important aspect of the RPP method is that it first utilizes a probe set level analysis to identify SFP-containing probe sets and then chooses individual probes from within each SFP-containing probe set. The net result is the identification of probes that directly overlay polymorphic sequences.

Separate comparisons were made between two genotypes (with two replicates each) for unstressed and drought stressed treatments, resulting in two SFP lists. In the context of SFPs, there is no necessity to have separate stress and control lists; in fact it would be simpler and less costly to have only one SFP list from highly complex RNA made by blending stressed and unstressed RNA, as in Cui *et al*. [20]. In our case, two separate lists were available as a consequence of another study not described here which compared gene expression patterns in stressed and control plants (data not shown). At 15% outlying score cut-off, we detected 488 SFP probes in stressed and 661 SFP probes in unstressed treatments. The union of these two lists contained 1058 SFP probes and the intersection contained 91. A total of 37 primer pairs targeting 37 putative SFP probe sets were initially tested, of which 25 yielded single amplicons of the expected sizes from both parents. These 25 amplicons targeted 14 probe sets selected from the intersection of the two SFP probe set lists and 11 from the remaining SFP probe sets. As shown in Additional file 1, 9 of the 14 SFP probe sets (64%) from the intersection list were validated at the DNA sequence level and 8 of the other 11 (73%) were validated.

### Genomic DNA isolation

Young leaves from one-week-old cowpea seedlings were ground with liquid nitrogen. Approximately ~0.1 g of ground tissue was used for genomic DNA isolation using a DNeasy Plant Mini Kit (Qiagen, USA) according to the manufacturer's recommendations. Eluted genomic DNA was examined by UV absorbance and with 1.0% agarose gel electrophoresis for quality and quantity evaluation.

### Primer design and PCR

The soybean genome array unigene sequences were used to query (using blastx) *Arabidopsis *translated gene models (version 7.0) from The *Arabidopsis *Information Resource (TAIR). Annotations for the Affymetrix soybean probe sets were compiled into a browser called HarvEST:SoyChip which can be accessed online or downloaded for Windows installation [39]. Cowpea ESTs available from GenBank were assembled using the CAP3 program [40] and compiled into a browser called HarvEST:Cowpea which can be accessed online or downloaded for Windows installation [41]. Cowpea methyl-filtered sequences were obtained from Dr. Michael Timko, Department of Biology, University of Virginia [42]. The soybean unigenes corresponding to each SFP probe set were used to query cowpea unigenes in HarvEST:Cowpea and cowpea methyl-filtered sequences. The cowpea sequences corresponding to SFPs were then used for PCR primer design in the flanking regions of SFP position. Primers were designed using Primer3 web version software [43]. The strategy used for primer design is illustrated in Additional file 2. All primer pairs were designed with T_m_~55°C.

PCR was performed in 20 μl reactions containing 20~25 ng of genomic DNA, 0.1 μM of primers, 0.2 mM dNTPs, and 1 unit of *Taq *DNA polymerase (New England Biolabs, USA). The reaction included an initial 5 min denaturation at 95°C, followed by 35 cycles of PCR (94°C, 30 sec; 55°C, 45 sec; 72°C, 1 min), and a final 5 min extension at 72°C. Aliquots (4 μl) of the PCR products were loaded and separated on 1.2% agarose gel by electrophoresis. A higher T_m _was used when non-specific bands were amplified. PCR products were purified using QIAquick PCR purification Kit (Qiagen, USA) after confirming their uniformity on agarose gels.

### DNA sequencing and analysis

DNA sequencing was performed using the dideoxynucleotide chain termination method [44]. Both strands of the amplified PCR products were sequenced with an ABI-PRISM 3730xl Autosequencer (Aplied Biosystems, USA) at the Core Instrumentation Facility of the UC Riverside Institute for Integrative Genome Biology. The forward and reverse sequence reads for each genotype for a particular primer pair were assembled using Contig Express (Invitrogen, USA) and the consensus sequence was used for alignment. These sequences were then compared with each soybean Sequence Information File (SIF, the target sequence which extends from the 5' end of the 5'-most probe to the 3' end of the 3'-most probe) and cowpea sequence using AlignX (Invitrogen, USA). Comparisons of nucleotide sequence similarity were displayed using GeneDoc [45]. Cowpea genomic amplicon sequences have been deposited into the dbGSS Data Library [GenBank: ET041523 to ET041556].

### Data Availability

All expression data are available through the Gene Expression Omnibus (GEO) under platform GPL 4592, Series GSE 10284.

## Authors' contributions

TJC and PAR designed the experiment. SD, PRB and CS performed the research. XC provided the statistical analysis. SW produced HarvEST:Cowpea and HarvEST:SoyChip including blastx and annotation of SFP probe sets. JDE provided the plant materials. PRB, SD and TJC wrote the paper. All co-authors provided inputs to improve the manuscript.

## Supplementary Material

Additional file 1 A full list of SFPs and their outlying scores. Click here for file

Additional file 2 **Strategy for primer design**. F is forward primer, R is reverse primer and solid line indicates SFP probe position. Click here for file

## References

[B1] Ehlers JD, Hall AE (1997). Cowpea (*Vigna unguiculata *L. Walp.). Field Crops Research.

[B2] Langyintuo AS, Lowenberg-DeBoer J, Faye M, Lambert D, Ibro G, Moussa B, Kergna A, Kushwaha S, Musa S, Ntoukam G (2003). Cowpea supply and demand in West Africa. Field Crops Research.

[B3] Bressani R, Singh SR, Rachie, KO (1985). Nutritive value of cowpea. Cowpea research, production, and utilization.

[B4] Tarawali SA, Singh BB, Gupta SC, Tabo R, Harris F, Nokoe S, Ferna'ndez-Rivera S, Bationo A, Manyong VM, Makinde K, Odion EC, Fatokun CA, Tarawali S, Singh BB, Kormawa PM, Tamo M (2002). Cowpea as a key factor for a new approach to integrated crop-livestock systems research in the dry savannas of West Africa. Challenges and opportunities for enhancing sustainable cowpea production.

[B5] Maréchal R, Macherpa JM, Stainer F (1978). Etude taxonomique d'un groupe complexe d'espèces de genres Phaseolus et *Vigna *(Papillionaceae) sur la base des données morphologiques et polliniques, traitées par l'analyse informatique. Boissiera.

[B6] Timko MP, Ehlers JD, Roberts PA, Kole C (2007). Cowpea. Genome Mapping and Molecular Breeding in Plants.

[B7] Kumar LS (1999). DNA markers in plant improvement: An overview. Biotechnology Advances.

[B8] Simpson J, Paredes-Lopez O (1999). Molecular markers for crop improvement. Molecular biotechnology for plant food production.

[B9] Ouédraogo JT, Maheshwari V, Berner DK, St-Pierre CA, Belzile F, Timko MP (2001). Identification of AFLP markers linked to resistance of cowpea (*Vigna unguiculata *L.) to parasitism by *Striga gesnerioides*. Theoretical and Applied Genetics.

[B10] Ouédraogo JT, Gowda BS, Jean M, Close TJ, Ehlers JD, Hall AE, Gillespie AG, Roberts PA, Ismail AM, Bruening G, Gepts P, Timko MP, Belzile FJ (2002). An improved genetic linkage map for cowpea (*Vigna unguiculata *L.) combining AFLP, RFLP, RAPD, biochemical markers and biological resistance traits. Genome.

[B11] Ouédraogo JT, Tignegre JB, Timko MP, Belzile FJ (2002). AFLP markers linked to resistance against *Striga gesnerioides *race 1 in cowpea (*Vigna unguiculata*). Genome.

[B12] Bukar O, Kong L, Singh BB, Murdock L, Ohm HW (2004). AFLP and AFLP-derived SCAR markers associated with *Striga gesnerioides *resistance in cowpea. Crop Science.

[B13] Fatokun CA, Young ND, Myers GO, Singh BB, Mohan Raj DR, Dashiell KE, Jackai LEN (1997). Molecular markers and genome mapping in cowpea. Advances in cowpea research.

[B14] Menéndez CM, Hall AE, Gepts P (1997). A genetic linkage map of cowpea (*Vigna unguiculata*) developed from a cross between two inbred, domesticated lines. Theoretical and Applied Genetics.

[B15] Gupta PK, Rustgi S (2004). Molecular markers from the transcribed/expressed region of the genome in higher plants. Functional and Integrative Genomics.

[B16] Hazen SP, Kay SA (2003). Gene arrays are not just for measuring gene expression. Trends in Plant Science.

[B17] Winzeler EA, Richards DR, Conway AR, Goldstein AL, Kalman S, McCullough MJ, McCusker JH, Stevens DA, Wodicka L, Lockhart DJ (1998). Direct allelic variation scanning of the yeast genome. Science.

[B18] Borevitz JO, Liang D, Plouffe D, Chang HS, Zhu T, Weigel D, Berry CC, Winzeler E, Chory J (2003). Large-scale identification of single-feature polymorphisms in complex genomes. Genome Research.

[B19] Kumar R, Qiu J, Joshi T, Valliyodan B, Xu D, Nguyen HT (2007). Single feature polymorphism discovery in rice. PLoS One.

[B20] Cui XP, Xu J, Asghar R, Condamine P, Svensson JT, Wanamaker S, Stein N, Roose M, Close TJ (2005). Detecting single-feature polymorphisms using oligonucleotide arrays and robustified projection pursuit. Bioinformatics.

[B21] Rostoks N, Borevitz JO, Hedley PE, Russell J, Mudie S, Morris J, Cardle L, Marshall DF, Waugh R (2005). Single-feature polymorphism discovery in the barley transcriptome. Genome Biology.

[B22] Ronald J, Akey JM, Whittle J, Smith EN, Yvert G, Kruglyak L (2005). Simultaneous genotyping, gene-expression measurement, and detection of allele-specific expression with oligonucleotide arrays. Genome Research.

[B23] West MAL, van Leeuwen H, Kozik A, Kliebenstein DJ, Doerge RW, St Clair DA, Michelmore RW (2006). High-density haplotyping with microarray-based expression and single feature polymorphism markers in Arabidopsis. Genome Research.

[B24] Kim S, Zhao KY, Jiang R, Molitor J, Borevitz JO, Nordborg M, Maijoram P (2006). Association mapping with single-feature polymorphisms. Genetics.

[B25] Jiang R, Marjoram P, Borevitz JO, Tavare S (2006). Inferring population parameters from single-feature polymorphism data. Genetics.

[B26] Naylor RL, Falcon WP, Goodman RM, Jahn MM, Sengooba T, Tefera H, Nelson RJ (2004). Biotechnology in the developing world: a case for increased investments in orphan crops. Food Policy.

[B27] Luo ZW, Potokina E, Druka A, Wise RP, Waugh R, Kearsey MJ (2007). SFP genotyping from Affymetrix arrays is robust but largely detects cis acting expression regulators. Genetics.

[B28] Bhat PR, Lukaszewski A, Cui XP, Xu J, Svensson JT, Wanamaker S, Waines JG, Close TJ (2007). Mapping translocation breakpoints using a wheat microarray. Nucleic Acids Research.

[B29] Mergaert P, Nikovics K, Kelemen Z, Maunoury N, Vaubert D, Kondorosi A, Kondorosi E (2003). A novel family in Medicago truncatula consisting of more than 300 nodule-specific genes coding for small, secreted polypeptides with conserved cysteine motifs. Plant Physiology.

[B30] Gilad Y, Rifkin SA, Bertone P, Gerstein M, White KP (2005). Multi-species microarrays reveal the effect of sequence divergence on gene expression profiles. Genome Research.

[B31] Gilad Y, Oshlack A, Smyth GK, Speed TP, White KP (2006). Expression profiling in primates reveals a rapid evolution of human transcription factors. Nature.

[B32] Voolstra C, Tautz D, Farbrother P, Eichinger L, Harr B (2007). Contrasting evolution of expression differences in the testis between species and subspecies of the house mouse. Genome Research.

[B33] Liao BY, Zhang JZ (2006). Evolutionary conservation of expression profiles between human and mouse orthologous genes. Molecular Biology and Evolution.

[B34] Sartor MA, Zorn AM, Schwanekamp JA, Halbleib D, Karyala S, Howell ML, Dean GE, Medvedovic M, Tomlinson CR (2006). A new method to remove hybridization bias for interspecies comparison of global gene expression profiles uncovers an association between mRNA sequence divergence and differential gene expression in Xenopus. Nucleic Acids Research.

[B35] Moehring AJ, Teeter KC, Noor MAF (2007). Genome-wide patterns of expression in Drosophila pure species and hybrid males. II. Examination of multiple-species hybridizations, platforms, and life cycle stages. Molecular Biology and Evolution.

[B36] Nieto-Diaz M, Pita-Thomas W, Nieto-Sampedro M (2007). Cross-species analysis of gene expression in non-model mammals: reproducibility of hybridization on high density oligonucleotide microarrays. Bmc Genomics.

[B37] Helms D, Panella L, Buddenhagen IW, Tucker CL, Gepts PL (1991). Registration of 'California Blackeye 46' cowpea. Crop Science.

[B38] Zhu HY, Choi HK, Cook DR, Shoemaker RC (2005). Bridging model and crop legumes through comparative genomics. Plant Physiology.

[B39] HarvEST:SoyChip. http://www.harvest-web.org.

[B40] Huang XQ, Madan A (1999). CAP3: A DNA sequence assembly program. Genome Research.

[B41] HarvEST:Cowpea. http://www.harvest-web.org.

[B42] Cowpea Genomics Initiative. http://cowpeagenomics.med.virginia.edu/.

[B43] Primer 3. http://frodo.wi.mit.edu/cgi-bin/primer3/primer3_www.cgi.

[B44] Sanger F, Nicklen S, Coulson AR (1977). DNA Sequencing with Chain-Terminating Inhibitors. Proceedings of the National Academy of Sciences of the United States of America.

[B45] Nicholas KB, Nicholas HBJ, Deerfield DW (1997). GeneDoc: analysis and visualization of genetic variation. EMBnet News.

