# Supplementary material for: Detection and validation of single feature polymorphisms in cowpea (Vigna unguiculata L. Walp) using a soybean genome array
Source: BMC Genomics. 2008 Feb 28;9:107. doi: 10.1186/1471-2164-9-107 (PMC2270837; doi:10.1186/1471-2164-9-107)
Supplement: Additional file 2 — Strategy for primer design. F is forward primer, R is reverse primer and solid line indicates SFP probe position. [file 1471-2164-9-107-S2.pdf]

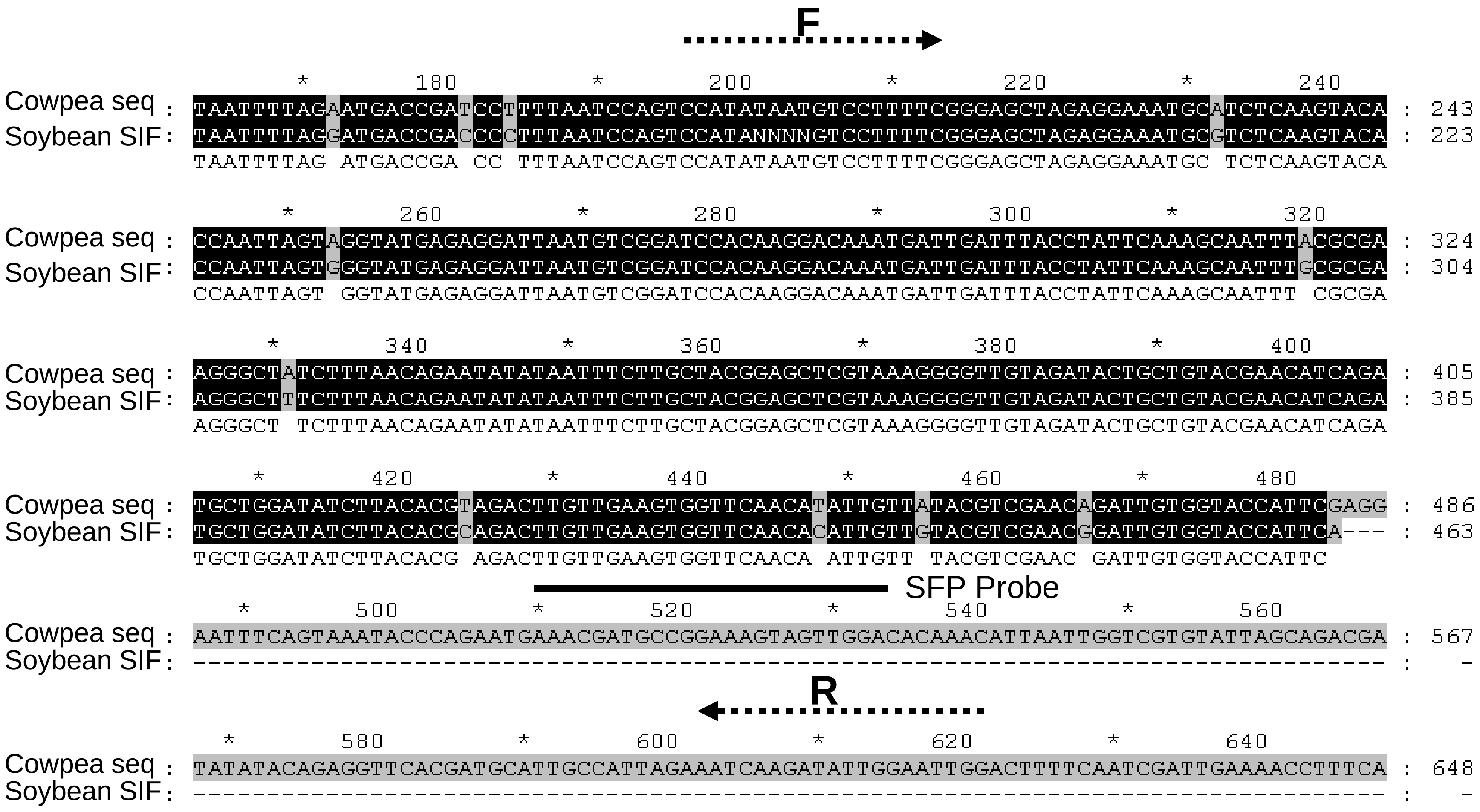

Additional File 2. Strategy for primer design. F is forward primer, R is reverse primer and solid line indicate SFP probe position.
